# SMARTWOMAN™: Feasibility assessment of a smartphone app to control cardiovascular risk factors in vulnerable diabetic women

**DOI:** 10.1002/clc.23124

**Published:** 2019-01-17

**Authors:** Nanette K. Wenger, Olubunmi O. Williams, Susmita Parashar

**Affiliations:** ^1^ Department of Medicine (Cardiology), Emory University School of Medicine, Emory Heart and Vascular Center Emory Women's Heart Center Atlanta Georgia; ^2^ Emory University School of Medicine Atlanta Georgia; ^3^ Department of Medicine (Cardiology), Emory University School of Medicine Emory Women's Heart Center Atlanta Georgia

**Keywords:** CV risk factors, smartphone app, vulnerable diabetic women

## Abstract

**Background/Hypothesis:**

SMARTWOMAN™ was designed to develop and assess the feasibility of a smartphone app to control cardiovascular risk factors in vulnerable diabetic women.

**Methods:**

Fourteen African‐American women with diabetes and without known cardiovascular disease were enrolled. A weight‐scale, glucometer, sphygmomanometer, and FitBit were synchronized to the smartphone, and text messaging was provided. Follow‐up was 6 months.

**Results:**

Patients were able to follow instructions for app use and device prompts. Weekly device reporting was 85% for blood glucose, 82.5% for daily steps, and 77% for systolic blood pressure. Patient engagement levels were 85% to 100% at 1 month and 50% to 78% at month 6. The majority reported text messages to be useful, easy to understand, and appropriate in frequency. The women indicated on the exit questionnaire that study participation increased their motivation and ability to take charge of their health.

**Conclusions:**

Use of a smartphone app to control cardiovascular risk factors appears feasible in a population of vulnerable indigent African‐American diabetic women, resulted in increased patient satisfaction and positive reinforcement to healthy behaviors, and warrants a larger clinical outcome trial.

## 1 BACKGROUND INFORMATION

Cardiovascular (CV) disease disparately affects women with diabetes mellitus, who have an excess risk of coronary heart disease (CHD).^1^ The relative risk for CHD is 50% higher in diabetic women than that in diabetic men, with a comparably higher risk of fatal CHD.^2^ Myocardial infarction (MI) mortality in diabetic women is greater than that in diabetic men. Diabetic women have a more adverse CV risk profile than their male peers and are less likely to have control of their CV risk factors. Furthermore, there are worse outcomes in subpopulations of diabetic women, in particular indigent women and those of racial and ethnic minorities, of lower socioeconomic status and lower educational status. Major gender disparities occur in the quality of preventive care for diabetes and CHD.^3^, ^4^ These data derive from commercial and Medicare plans,^5^, ^6^ and unfortunately data for uninsured women, the most vulnerable, have not been analyzed.

The age at the diagnosis of diabetes is decreasing in women, likely related to the obesity epidemic, raising the question as to whether a progressively longer duration of diabetes will further accelerate the adverse clinical CV outcomes in younger women.

## 2 GENERAL PRINCIPLES: PATIENT SELF‐MANAGEMENT TOOLS

Patient self‐management tools refer to technologies used by consumers (patients) to manage health issues, particularly chronic disease such as diabetes mellitus. They are designed to facilitate autonomy in self‐management, to augment patient/clinician collaboration and communication, and to enhance patient empowerment.^7^ Digital health, or eHealth, is the use of emerging internet communication and information technologies to improve healthcare and health behaviors.^8^ Mobile health or mHealth, a subsegment of eHealth additionally uses mobile computing and communication technologies (eg, mobile phones and wearable sensors) for patient self‐management. Typically, these products present reminder messages or prompts and deliver basic questions that encourage interaction or input of information that mimic an experience with a clinician. Many incorporate clinical decision support, and many provide links to health education materials, thus enabling health decision‐making and encouraging positive health behaviors.

Smartphones have revolutionized the communication landscape and have the potential to increase access to health information and preventive care for uninsured patients^9^, those living in rural areas, immigrants, and the elderly. Eighty‐five percent of U.S. adults own a cell phone, and 50.2% have a smartphone.^10^ Approximately 61% of blacks own a smartphone.^8^ One half of smartphone owners use them to get health information and, as of 2012, 1/5 of smartphone owners have health applications (apps) on their devices. One limitation is that solely text messaging offers limited interaction; several reports have described additional benefit with synchronized attachments and automatic data input. In addition, there are concerns that the health‐promoting smartphone apps may fail to incorporate contemporary evidence‐based content. Literature is also very limited regarding data on the health‐related mobile technologies focused specifically on CVD prevention.

Research is needed regarding the validity and efficacy of these devices and apps. Even more limited are data on indigent patients with poor healthcare resources. In the absence of such data, clinicians may be hesitant to recommend or endorse any programs to their patients, thus missing an opportunity to improve their engagement in healthy behaviors. Of interest is that the National Health Service in the United Kingdom both lists and recommends selected apps. The U.S. Food and Drug Administration has raised concern about device usability, accessibility, readability, health literacy level, device connectivity, security, and user privacy; a patient‐centered design was recommended by FDA Regulation 21CSR839.30. The 2012 FDA Safety and Innovation Act (FDASIA) recommended the creation of a health IT safety center, but such does not currently exist.

The objective of our pilot study was to develop and examine the feasibility of mobile health to improve health behaviors and self‐management regarding coronary risk factors in indigent women with diabetes.

## 3 SMARTWOMAN™ PILOT STUDY DESIGN

### 3.1 Participants

Our study population consisted of women with diabetes without known CHD at an inner city indigent care public hospital (Grady Memorial Hospital in Atlanta, Georgia). These women were referred by their primary care physicians for poorly controlled diabetes to a specialized diabetes clinic. They had suboptimal access to care, lack of transportation and resources to attend hospital or clinics, and often had low healthy literacy levels. We included patients who were compliant with clinic visits and were interested in improving their health. The study design and informed consent document were approved by the Emory University School of Medicine Institutional Review Board (IRB).

A smartphone was provided to each participant for the duration of the study by the Verizon Foundation. Communication‐enabled measurement devices were provided to synchronize with the smartphone: a weight scale, a glucometer, and a sphygmomanometer were provided by Ideal Life, and a FitBit was provided by the Verizon Foundation. The smartphone application development was undertaken in conjunction with CircleLink Health. Funding for the study was provided by the Society for Women's Health Research/Verizon Foundation.

An exit questionnaire (see Supporting Information 3 S1) was designed to ascertain the usefulness of text messaging for checking blood glucose, blood pressure, weight, daily steps, smoking cessation; taking insulin or oral diabetes medication, blood pressure or cholesterol medication; and following a diabetic, healthful diet. Patients were queried about the most and least useful aspects of the text messaging, whether they were disruptive, as well as how often they would prefer to receive text messaging, time of day, and so on. They were queried about the website links sent by text messages, whether study participation encouraged self‐management and understanding about their health condition and whether the FitBit helped their activity level. They were queried about the devices provided—which they liked best and least and whether adequate technical support was provided for using the devices.

## 4 DEVELOPMENT OF THE SMARTPHONE APP

The content base for the smartphone app was the American Heart Association's “Life's Simple 7” prevention module^11^ with its core elements individualized to maximize patient engagement, that is, only those risk attributes relative to an individual woman were included in that patient's communication base. The content used nationally accepted patient risk factor goals. We further tailored our app to our target population by previously performing a community needs assessment by conducting a focus group of women similar to our participant population regarding the value, need, interest and least intrusive methods of participant engagement.

Mobile technology was integrated into the overall program, with communication enabled devices (weight scales, glucometers, and sphygmomanometers) synchronized to the smartphone. A patient‐based FitBit was used to monitor activity level.

Design of the resource material used existing credible publicly available documents for patient information and education, such as the web‐based CardioSmart. The goal of the app design was to minimize patient intrusion while maximizing patient data collection. As such, during the course of the study, the frequency of selected metrics that had poor response rates was decreased to improve the data set.

Interactive patient contacts were designed to maximize patient participation and to reinforce availability of help. The content was serially simplified, compatible with resource interactions.

The goal was for engagement, first monitoring the data so that the clinician could intervene for emergency alert metrics, for example, dangerously high or low levels of blood glucose or blood pressure. Nonparticipating patients were identified early, with interventions undertaken to increase their engagement. There was consistent smartphone feedback regarding progress to goals for individual risk factors (Table 1).

**Table 1:** Sample question template: Blood pressure (BP)

Did you take your blood pressure medicine today? Y or N
Y = good. Keep taking your medicine. It is important part of keeping healthy N = taking your medicine is a key part of your treatment. Did you forget, lose medicine, run out? If you have questions or concerns about your medicine, please respond with Y and we will notify the team.
We have not received a BP reading from you this week. If you need someone to call you, please type Y or N
Y = thanks. We will notify the coordinator N = please remember it is important to measure your BP. Please use your BP device as soon as you can
Thanks for sending us your BP
Goal: If at goal = congratulations. Your BP is where it should be.
Keep up the good work and remember to check your BP at the same time each week
Near goal: Not at goal but better than last time. Your BP is improving, so keep working a it and you will reach your goal. Remember to check your BP at the same time each week. Above goal: Not at goal and not as good as last week. Remember diet and exercise will help you reduce your BP. Click here to get some more information. http://www.cardiosmart.org/Healthwise/2p26/24/2p2624

The execution model, as described, refined the messaging and adjusted the program to eliminate suboptimal messages and content. It was designed to easily access clinical and engagement data. There was serial monitoring regarding reasonable characteristics of the data, that is, to check outliers. In this way, we ascertained that the data from the weight scales seemed unreliable in that in some instances likely someone other than the patient in the household used the weight scale that was synchronized to the smartphone.

A portal was designed for easy access both to individual patient and to population data. Increased intervention over time by CircleLink Health staff facilitators improved patient access and resolved device synchronization problems (Table 2).

**Table 2:** Sample telephone script: Exercise

**[if no FitBit data or sporadic data:]**
I see that you have not been wearing your FitBit daily. Can you tell us why you have been unable to wear the FitBit daily?
**[if they have been wearing their FitBit, but report low numbers or not improving:]**
You have been wearing your FitBit. Great job! However, I see that the number of steps you are walking [is very low/has not increased.] can you tell me why? [try to understand if they are wearing but not active, or forgetting to wear it sometimes]
What is the greatest barrier to syncing the FitBit daily?
Did you find it helpful to receive a weekly text reminder to charge and sync your FitBit, and wear it daily?
Yes
No
Why did you or did you not find it helpful?
Did you find it helpful to receive a weekly text reminder to increase your number of steps walked daily?
Yes
No
Why did you or did you not find it helpful?
Did the research coordinator explain how to use the FitBit, including how to charge it and how to sync it with your phone?
Yes
No
Did she explain why it was important to wear the FitBit daily
Yes
No

We initially conducted a pilot study utilizing volunteer cardiology fellows in training to test the ability of the smartphone to synchronize with the devices, and to ascertain the user‐friendliness of the apps.

The study coordinator paired each device and FitBit with the smartphone that was provided to the participant. All devices were Bluetooth enabled to facilitate pairing. Devices were tested to ensure that they were synchronizing and transmitting measurements/data that participants provided to the smartphone. CircleLink Health study staff tracked the data on the devices. The study coordinator instructed each participant in the use of the smartphone, FitBit, and each device. Participants were also provided with the study coordinator's telephone number for questions or troubleshooting.

Participants were instructed to measure blood glucose every morning, blood pressure and weight weekly (on the same day), and to wear the FitBit at all times. They were instructed that they could remove the FitBit and charge it at night while they slept.

Outcome measurements were blood pressure, blood glucose, weight, and daily steps. We also assessed patient engagement to messaging prompts. When data received appeared to be suboptimal, prompts and text messages were developed, with tips as to how to better respond.

## 5 RESULTS

Participant characteristics: fourteen African‐American women with diabetes were enrolled in the study. Their mean age was 52 years, with an age range of 34 to 68 years. Two of the 14 were cigarette smokers, 12 had hypertension, 11 had hypercholesterolemia, 12 were obese, and nine were not employed outside the home.

Although not used as outcome measures for our study, the overall group metrics for systolic blood pressure (mm Hg) were 131 at baseline and 124 at 6‐month study end. Random blood glucose (mg/dL) was 180 at baseline and 110 at 6 month study end. The daily steps were 3400 at baseline and 3855 at study end.

The overall weekly device reporting was 85% for blood glucose, 82.5% for daily steps, and 77% for systolic blood pressure. Daily steps were adjusted at baseline for the two patients who initially had a very high level of activity but rapidly regressed to the average of the group.

Patient engagement levels (which were assessed by device use, provision of data, responses to text messaging prompts) for month 1 varied from 85% to 100% and at month 6 was 50% to 78% (Table 3). The response rate to questions was 60% to 95% for medication questions, 35% for the dietary question related to vegetables, and 75% to 98% for other dietary and smoking questions.

**Table 3:** Feasibility assessment (using devices and responding to text messages)

Category	Percent response
Overall weekly device reporting	
Blood glucose	85%
Daily steps	82.5%
Systolic blood pressure	77%
Patient engagement (device use and response to text messages)	
Medications	60%‐95%
Dietary vegetables	35%
Other dietary and smoking questions.	75%‐98%
Overall patient engagement	
Month 1	Month 6
85%‐100%	50%‐78%

## 6 EXIT QUESTIONNAIRE

Based on the exit questionnaire, patients perceived that it was useful to receive text message prompts to check blood glucose, blood pressure, weight, and daily steps (see Supporting Information 3 S1). They found the prompts to take medications useful, as were the tips for a healthier diet and adherence to a diabetic diet. The participants found useful the text messages for blood glucose being too high or too low, and followed the provided suggestions. The weekly summary of device use was also found helpful. The FitBit app helped patients track activity levels and either made them more active or to consciously try to be active.

The majority reported the text messages to be useful (92%), easy to understand (91%), and appropriate in frequency (92%). None found the text messages disruptive or too frequent. Participants found positive text messages as a strong positive reinforcement to healthy behaviors. Some of the comments included “interesting journey—reminders valuable without being naggy,” “liked being told doing a good job.”

The women indicated that study participation increased their motivation to take charge of their health, as well as providing an increased understanding of their health condition and personal healthcare. Adequate technical support was reported throughout the study, “felt as if I was communicating with a person.” Patients reported they would probably use another health‐related app in the future and would consider purchasing one depending on the price.

## 7 DISCUSSION

Our pilot study suggests that this vulnerable population of diabetic women was interested in improving their personal health. They were able to follow app use instructions and device synchronization after initial troubleshooting and were receptive to telephone calls and smartphone prompts via text messages.

The aphorism “less is more” was very applicable, in that there were increased responses with decreased frequency of the questions asked. We have learned that the app should be on a personal smartphone in future studies, in contrast to the separate unit used in this research study, which was often left at home and used only at selected evening or weekend hours.

Personal interaction with a facilitator appeared preferable, compared with isolated smartphone use. The use of Bluetooth enabled devices which automatically synchronized with the smartphone was probably an advantage in data collection (or capture) as study participants did not have to manually upload data creating an additional step in the process.

## 8 SUMMARY

Use of a smartphone app to control CV risk factors appears feasible in a population of vulnerable indigent African‐American diabetic women. They were interested in and able to use the apps, which resulted in increased patient satisfaction and positive reinforcement to healthy behaviors. This warrants a larger clinical outcome trial.

## CONFLICT OF INTEREST

The authors declare no potential conflict of interests.

## Supporting information


**Appendix S1.** Questionnaire for SMARTWOMAN
