# Supplementary material for: SMARTWOMAN™: Feasibility assessment of a smartphone app to control cardiovascular risk factors in vulnerable diabetic women
Source: Clin Cardiol. 2019 Jan 17;42(2):217–21. doi: 10.1002/clc.23124 (PMC6712310; doi:10.1002/clc.23124)
Supplement: Supplementary file 1 — Appendix S1. Questionnaire for SMARTWOMAN [file CLC-42-217-s001.docx]

**Appendix: Questionnaire for SMARTWOMAN^TM^**

1. Did you find it useful to get the text messages about checking your blood glucose?

___ Yes ___ No Comments? _______________________

1. Did you find it useful to get the text messages about checking your BP?

___ Yes ___ No Comments? _________________________

1. Did you find it useful to get the text messages about checking your weight?

___ Yes ___ No Comments? _________________________

1. Did you find it useful to get the text messages about number of steps taken every day (or week)? ___ Yes ___ No Comments? _________________________
2. Did you find it useful to get text messages encouraging you to try to stop smoking (if applicable to you)? ___ Yes ___ No Comments? _________________________
3. Did you find it useful to get the text messages reminding you to take your insulin (if applicable to you)? ___ Yes ___ No Comments? _________________________
4. Did you find it useful to get the text messages reminding you to take your oral diabetes medication, e.g. metformin (if applicable to you)? ___ Yes ___ No Comments? _________________________
5. Did you find it useful to get the text messages reminding you to take your blood pressure medication (if applicable to you)? ___ Yes ___ No Comments? ___________________
6. Did you find it useful to get the text messages reminding you to take your cholesterol medication (if applicable to you)? ___ Yes ___ No Comments? ___________________
7. Did you find it useful to get text messages encouraging you to follow a diabetic diet?

___ Yes ___ No Comments? _________________________

1. Did you find text messages about tips for a healthier diet helpful? ___ Yes ___ No Comments? _________________________
2. What did you find most useful about the text messages?
3. What did you find least useful about the text messages?
4. Did you find it disruptive to receive text messages? ___ Yes ___ No Comments? _________________________
5. Do you think the text messages were too frequent? ___ Yes ___ No Comments? _________________________
6. If yes, how often would you prefer to receive text messages? ___ Weekly

__ Once every two weeks ___ Monthly ___ Other ___________________

1. At what time of day would you prefer to receive text messages? ___ Morning

___ Afternoon ___ Night __ No preference ___ Other __________________

1. How often do you think you responded to text messages? __ Less than half the time

___ About half the time ___ Most of the time ___ All the time

1. Did you ever get a text message telling you that your blood glucose was ___ too high

___ too low ___ both?

___ Yes ___ No

1. Was it helpful to get that message? ___ Yes ___ No Comments? _________________
2. If no, why not ________________________ E.G., I already knew what to do.
3. If yes, did you follow the suggestions/instructions? ___ Yes ___ No
4. If no, why not? ______________________________ E.g., I already knew what to do.
5. Did you ever look at the weekly summary from your use of the devices (you should have received a text message with a link to follow to see your activity every Monday morning)

___ Yes ___ No Comments? _________________

1. If yes, did you find it useful? ___ Yes ___ No Comments? _________________
2. If no, why didn’t you look at the summary? ___ I didn’t want to ___ I didn’t know it was there ___ Other
3. If you said you didn’t know it was there in the previous question, would you have looked at it if you knew it was there? ___ Yes ___ No Comments? _________________
4. Did you ever follow the website links that were periodically sent to you by test message?

___ Yes ___ No Comments? _________________

1. If yes, did you find it useful? ___ Yes ___ No Comments? _________________
2. If no, why didn’t you look at the summary? ___ I didn’t want to ___ I didn’t know it was there ___Other______________________
3. Did participating in this study make you feel more in charge about taking care of your health? ___ Yes ___ No Comments? _________________
4. Do you feel being a participant in this study will encourage you to use other health-related apps? ___ Yes ___ No Comments? _________________
5. Has being in this study increased your understanding about your health condition?

___ Yes ___ No Comments? ________________

1. Has being in this study increased your understanding of how to take care of your health:

___ Yes ___ No Comments? ________________

1. How often do you think you remembered to wear your fitbit? ­­­___ Less than half of the time ___ About half of the time ___Most of the time ___All of the time
2. If you didn’t wear the fitbit all the time, why not? ___ I forgot to wear it ___ I didn’t like wearing it ___ I didn’t think I moved around/walked enough to wear it all the time ___Other ___________________________________
3. Do you think wearing the fitbit helped you to be more active? ___ Yes ___ No Comments? ________________
4. Do you think wearing the fitbit made you consciously try to be more active? ___ Yes ___ No Comments? ________________
5. Did you ever check the fitbit app on your phone to track your activity, e.g., number of steps walked or number of stairs climbed? ___ Yes ___ No
6. What did you like best about using the devices you were provided with
   1. Glucometer
   2. BP machine
   3. Scale
   4. Fitbit
7. What did you like least about using the devices you were provided with
   1. Glucometer
   2. BP machine
   3. Scale
   4. Fitbit
8. Did you feel you got enough technical support while using the devices? ___ Yes ___ No Comments? ________________
9. Would you have preferred to receive a customer support number from the beginning?

___ Yes ___ No Comments? ________________

1. If there was a similar app available to monitor your blood glucose, BP, weight, and activity, would you use it? ___ Yes ___ No Comments? ________________
2. If it was not free, would you buy it/pay for it from the app store on your phone (Apple or Android)? ___ Yes ___ No Comments? ________________
3. Do you have any comments about your participation in the study? ___ Yes ___ No Comments? ________________
